# Percutaneous Deep Venous Arterialization for Limb Salvage in No Option Patients with Chronic Limb-Threatening Ischemia

**DOI:** 10.3390/jcm12237324

**Published:** 2023-11-26

**Authors:** Stavros Spiliopoulos, Efstathia Davoutis, Nikolaos-Achilleas Arkoudis, Kaji Sritharan, Symeon Lechareas

**Affiliations:** 1 2nd Department of Radiology, Interventional Radiology Unit, Medical School, National and Kapodistrian University of Athens, “Attikon” University General Hospital, 15784 Athens, Greece; davoutis@gmail.com (E.D.); nick.arkoudis@gmail.com (N.-A.A.); 2 Department of Vascular Surgery, Liverpool University NHS Foundation Trust, Liverpool L69 3BX, UK; kajisritharan@yahoo.co.uk; 3 Department of Interventional Radiology, Liverpool University NHS Foundation Trust, Liverpool L69 3BX, UK; slehareas@gmail.com

**Keywords:** chronic limb-threatening ischemia, percutaneous deep venous arterialization, endovascular treatment, desert foot

## Abstract

An endovascular approach is often considered the first line treatment option for lower limb chronic limb-threatening ischemia (CLTI), which is defined by the presence of ischemic rest pain and severe tissue loss, such as ulcers or gangrene. Although the technical success rate of endovascular revascularization is high, in specific patients with advanced infrapopliteal disease and the absence of run-off tibial vessels, the so-called ‘desert foot’, the chance of successful endovascular revascularization is minimal. In order to avoid primary amputation, several treatment options are currently being investigated, including gene therapy and deep venous arterialization. This review focuses on the percutaneous deep venous arterialization technique as a promising, minimally invasive treatment option for limb salvage in CLTI patients presenting with a ‘desert foot’.

## 1. Introduction

Peripheral artery disease (PAD) is a progressive disease caused mainly by atherosclerosis and characterized by the stenosis and/or occlusion of the arteries, with the lower extremities more commonly affected than the upper extremities [1]. Smoking, diabetes mellitus, obesity (characterized by a body mass index exceeding 30), advancing age, hypertension, elevated cholesterol levels, increased homocysteine levels, and a family history of cardiovascular disease are risk factors for atherosclerosis. The development of PAD diminishes the blood flow to the distal extremities, thus restricting tissue perfusion [2,3]. End-stage PAD often presents with chronic limb-threatening ischemia (CLTI), defined by the presence of ischemic rest pain and severe tissue loss, such as ulcers or gangrene, according to the Inter-Society Consensus for the Management of Peripheral Arterial Disease (TASC II 2007) [4]. An all-cause mortality rate of 22% has been reported within 12 months of diagnosis, related mostly to cardiovascular and peripheral complications (i.e., infections) [5,6,7].

Therefore, effective PAD management tailored to the patient is essential for improving long-term survival and reduce morbidity. Aggressive risk factor control improves cardiovascular outcomes and disease-specific treatment options include lifestyle changes, pharmacotherapy, exercise therapy, and endovascular or surgical interventions [8].

According to two published meta-analyses, the rate of limb salvage is similar for both an endovascular and open surgical approaches [7,8]. However, since both methods demonstrate specific advantages and disadvantages, patient selection based on multiple clinical and morphological characteristics is imperative for clinical success. Regardless of the method of revascularization used, the main goal of revascularization is to create adequate arterial perfusion to the diseased distal foot to enable wound healing [6]. In patients with multiple comorbidities or a low chance of successful revascularization due to advanced disease in the tibial arteries, pedal arteries, or in the small arteries in the foot (otherwise known as ‘desert foot’), there are often no options available for successful revascularization, leading to primary amputation being considered the only solution. Patients with small artery disease (SAD) are typically those with diabetes, end-stage renal disease, and thromboangiitis obliterans, and are estimated to account for around 20% of patients with PAD. Moreover, their prognosis, including a 20% mortality at 6 months, is noted to be worse than those with less severe disease [2,9,10].

Alternative treatment options for patients with no-option CLTI have been described, and these include spinal cord stimulation, lumbar sympathectomy, intermittent pneumatic compression, hyperbaric oxygen therapy, stem cell therapy, and prostanoid therapy. However, the outcomes of these approaches have been poor in trials [9,10]. For this group of patients, deep venous arterialization (DVA) has, however, been reported as a promising alternative to amputation. 

### Interpreting DVA

Adequate tissue perfusion is of paramount importance to avoid amputations. The principle underlying DVA is that the perfusion of the peripheral ischemic tissues and the plantar venous arch is achieved through the arterialization of the disease-free venous bed by creating an arterial-deep venous shunt that enables wound healing [10]. Femoral arteriovenous anastomosis was first described in dogs in 1881 and in humans in 1894 by Francois-Frank, with poor rates of wound healing and limb salvage and several complications [11,12]. Several studies have since been published investigating the technique, but after 1916, interest in the field diminished [13]. Later in 1977, the modern version of the procedure was introduced by Sheil, who described the anastomosis of an arterialized great saphenous vein to the dorsal venous arch of the foot [14]. Potential mechanisms of action for this procedure include reversed blood flow in the arterioles and capillaries permitting tissue nutrition and neovascularization stimulated by angiogenic factors, leading to a remodeling of the vascular distribution system of the foot [15,16,17].

There are various ways in which DVA can be performed, including surgical, endovascular, or hybrid approaches. Surgical DVA was pioneered by Herb Dardik. The proximal anastomosis of the great saphenous vein (GSV) and the most distal patent artery (popliteal artery or superficial femoral artery) is performed [18]. Limb salvage for surgical DVA has been reported in the literature to be 70% [19]. The evolution of endovascular technology has allowed for the development of percutaneous approaches, and interest in this approach has increased as this less invasive approach reduces the burden on the multiple comorbidities that are often present in patients with no-option CLTI.

The purpose of this review is to present the technique of DVA, focusing on a percutaneous approach; to analyze how it is performed; and to review the most up-to-date data on its effectiveness.

## 2. Percutaneous DVA (pDVA)

### 2.1. Benefits of pDVA

The lack of options for end-stage CLTI patients highlights the importance of pDVA as an option for limb salvage when amputation seems to be the only option remaining. The major advantage of this approach is that it offers a minimally invasive option for patients with adequate endoluminal access. This, in turn, leads to lower periprocedural risks and the absence of wound creation, which is additionally important in an already ischemic leg [20].

### 2.2. Adverse Effects and Disadvantages of pDVA

As with any percutaneous catheterization, possible adverse effects of pDVA include vascular complications at the access site (i.e., bleeding events, hematoma), arterial and venous thromboembolic events (myocardial infarction, stroke, pulmonary embolism, etc.), contrast-induced nephropathy, or renal failure [20]. Apart from this, stent placement between the arterial and venous systems may lead to complications, such as thrombosis, circuit stenosis, and vessel spasm. 

The advantages and adverse effects of pDVA are summarized in Table 1.

### 2.3. pDVA Techniques

Different methods are used to perform pDVA, but in each method, there are three important steps: firstly, the formation of an arterio-venous (AV) fistula; secondly, disruption of venous valves so that flow reversal is possible; and thirdly, the prevention of the shunting of arterial blood through the AV fistula and other interconnecting veins. For the creation of an AV fistula, re-entry devices can be used as well as catheters, techniques involving snares (the so-called ‘venous arterialization simplified technique’; VAST), or double punctures (‘AV spear’) [21,22,23]. However, up to 20% of these procedures can fail because of the inability to create an AV communication. For instance, when the vessel wall is heavily calcified, the wire tip or needle cannot penetrate when a penetration wire or reentry device is used [24,25]. The LimFlow system (LimFlow SA, Paris, France) is currently the only registered device with which a pDVA procedure can be performed in its entirety, overcoming the challenge of AV fistula creation. However, it may increase the overall procedure cost, and regulatory approval is still pending in several markets [26].

### 2.4. Selection Criteria for pDVA Intervention

DVA is advocated in Rutherford class 5 or 6 patients who lack a distal target vessel for bypass or endovascular intervention and who have at least one patent tibial artery in the proximal segment [14]. As reported by Kum et al., general factors, angiographic factors, and wound-related factors should be the three focus areas in order to select patients suitable for undergoing pDVA [26]. In particular, concerning general factors, patients with poor cardiac function, pre-existing coronary lesions, and high output fistulas for dialysis access should be excluded. The same is true for patients with a history of deep venous thrombosis (DVT). With regard to angiographic factors, it is important to ensure the suitability of the tibial vessel used for crossing as well as the patency of the ipsilateral iliac and femoropopliteal arteries, in order to optimize inflow. Finally, in terms of the wound, patients with acute limb ischemia, systemic sepsis, and wounds around the distal retrograde access site should be considered unsuitable.

### 2.5. DVA Techniques

Several described methodologies for DVA have been described in the literature, though there has not been much standardization of procedural steps outside of the LimFlow trials. Techniques include a combination of open surgical, hybrid, and fully percutaneous approaches. Results across all techniques have been promising, demonstrating the feasibility of the arterialization of deep veins for restoring blood flow to the lower limb and potentially preventing major amputation. Open surgical DVA is achieved by pairing a surgical AV bypass with the physical effacement of the valves of the pedal loop vein. This technique has been shown to have comparable patency and limb salvage rates to surgical pedal bypass [27]. Hybrid superficial venous arterialization involves making an in situ open surgical anastomosis from the greater saphenous vein to the popliteal artery, followed by valve lysis and the distal endovascular focalization of flow [28,29]. Additionally, a single-stage approach has been described which involves open bypass with a stent to the venous outflow followed by percutaneous valve lysis [30]. A study conducted by Montelione et al. assessed patients undergoing the surgical arterialization of the deep venous circulation, followed by the endovascular selective embolization of venous escape routes from the foot. They found that this approach was a viable and efficient solution for preserving limbs in patients suffering from CLTI when no other options were available [31].

Finally, the fully percutaneous approach utilizes the tibial arteries as the donor vessel to create an AV crossing into the tibial vein, which is lined with a covered stent to focalize blood flow into the pedal veins, the valves of which are effaced percutaneously. pDVA can be performed using off-the-shelf devices or the proprietary LimFlow System, which is commercially available in Europe and the United States.

### 2.6. Benefits of LimFlow System—A Purpose-Built Device

The LimFlow System consists of a series of purpose-built devices, including crossing catheters for the creation of an AV fistula, a pushing valvulotome, and tapered and straight stent grafts. The arterial crossing catheter has a needle with a 10 mm reach that is deployed through the arterial wall into a 6 mm self-expanding nitinol basket deployed in the paired vein. This radiopaque target allows for easy AV alignment and wire capture for the AV crossing. The pushing valvulotome lyses the valves within the veins below the crossover point and distally into the pedal venous loop, allowing forward blood flow. Utilizing the valvulotome reduces the trauma to the vessel wall, avoiding trauma-induced stenosis in the outflow veins that can occur if using a balloon to efface the valves [29]. The tapered crossing stent allows for optimal sizing for both the artery and vein, and the straight extension stents prevent smaller veins from stealing flow back up the leg, focalizing the blood flow into the foot.

### 2.7. The pDVA Procedure Using the LimFlow System

**Figure 1:**
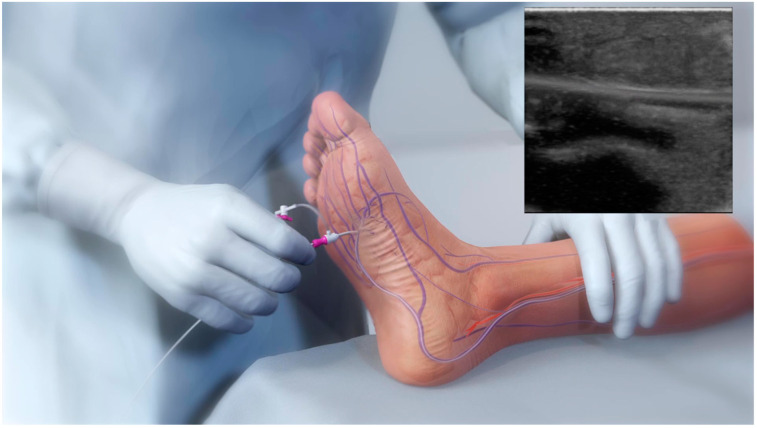
Retrograde lateral plantar vein access enabling the insertion of the venous catheter during the pDVA technique.

**Figure 2:**
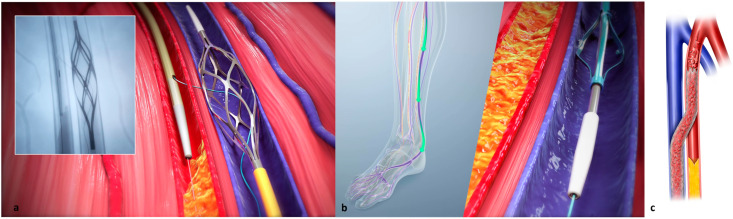
Illustration of pDVA procedural steps. (**a**) Advancing the needle from the arterial crossing catheter into the basket of the venous catheter to create the fistula. (**b**) Use of a push valvulotome to allow reversal flow in the vein and (**c**) final stent graft deployment for arteriovenous bridging.

Venous access is achieved in the lateral plantar vein (Figure 1), and the venous catheter is advanced into the target vein to the level of the intended crossing point into the tibial artery. Using simultaneous digital subtraction angiography (DSA) and venography, the shortest distance between the vessels is identified. An arterial crossing catheter is inserted via an antegrade femoral arterial approach and advanced distally into the artery to the below-the-knee crossing point. When the two catheters are aligned, artery-to-vein crossing is achieved by advancing the needle from the arterial crossing catheter into the basket of the venous catheter to achieve through-and-through wire access. The AV connection is then dilated using a small angioplasty balloon, allowing the passage of other devices. A 4F over-the-wire push valvulotome is then deployed and serves to render the venous valves insufficient, which allows the reversal of flow in the vein, thus directing arterial oxygenated blood into the foot. Self-expanding stent-grafts are then placed from the level of the ankle to just below the crossing point, creating a conduit for continuous blood flow and covering venous collaterals. Finally, a tapered, self-expanding, covered stent graft is deployed to optimize the transition and flow from the artery into the vein, preventing cardiac overload as well as leakage at the crossover point. Figure 2 illustrates the basic procedural steps. Procedural time ranges from 90 min to 4 h. After the procedure, patients should be maintained on lifelong antiplatelet therapy (oral clopidogrel or aspirin) in combination with warfarin or direct oral anticoagulants for 3 months. A follow-up protocol for monitoring blood flow into the foot is necessary using duplex ultrasound [32]. The LimFlow system earned a CE mark in 2016 and FDA approval in 2023, and is available in Europe and the United States.

Notably, an important observation that should be taken into consideration when using the LimFlow device is that prior to the creation of the AV fistula, patients may require an arterial and/or venous intervention (i.e., an arterioplasty, venoplasty) in order to optimize the DVA inflow and outflow vessels [26].

## 3. Studies Involving the LimFlow Device

### 3.1. PROMISE I

PROMISE I is a single-arm, multicenter pilot study and the first published report concerning the safety and efficacy of the LimFlow system in the United States. Thirty-two ‘no option’ CLTI patients were enrolled. Technical success was reported at 97%. Reinterventions were performed in 52% of patients, with most of them (88%) occurring within the first 3 months. The wound healing status of fully healed or healing was 67% and 75% at 6 and 12 months, respectively. The amputation-free survival rates (AFS) at 30 days, 6 months, and a year following the procedure were 91%, 74%, and 70%, respectively [33].

### 3.2. PROMISE II 

PROMISE II is a multi-center, prospective, single-arm study for the assessment of the safety and efficacy of pDVA using the LimFlow system. It encompassed 105 ‘no-option’ CLTI patients treated in the USA. The primary endpoint was AFS at 6 months. Secondary outcomes included the healing of chronic wounds caused by ischemia. The reported technical success rate was 99%. The AFS at 6 months was 66.1%. Limb salvage (the avoidance of above-ankle amputation) was attained in 67 patients (76.0% by Kaplan–Meier analysis). Complete wound healing occurred in 25% of patients, and 51% were in the process of healing [34].

### 3.3. ALPS

The ALPS study is a multi-center study that aimed to retrospectively evaluate 32 ‘no-option’ CLTI patients treated with the LimFlow system. The primary endpoint evaluated was AFS at 6 months, while secondary outcomes were wound healing, survival rate, and limb salvage at 6, 12, and 24 months. Technical success was achieved in 96.9% of cases. AFS at 6, 12, and 24 months was 83.9%, 71%, and 67.2%, respectively. Limb salvage at 6, 12, and 24 months was 86.8%, 79.8%, and 79.8%, respectively, and complete wound healing occurred in 36.6%, 68.2%, and 72.7% at 6, 12, and 24 months, respectively. Seventeen patients required reintervention for occlusion [35].

### 3.4. PROMISE International

PROMISE International is a single-arm, open-label, prospective study with a twelve-month follow-up period. It initially set out to enroll 50 ‘no-option’ CLTI patients but actual enrollment included 35 patients (NCT03321552). The primary endpoint are AFS, with secondary outcomes of complete wound healing, primary and secondary stent graft patency, limb salvage, renal function, and technical and procedural success [20]. The study has not yet reported its findings.

### 3.5. PROMISE UK

PROMISE UK (NCT03807661) is a multicenter post-market national trial that has completed recruitment and enrolled 28 patients but not reported its findings. Primary outcome was AFS at 1 year, and secondary outcomes include wound healing, primary and secondary stent graft patency, limb salvage, procedural and technical success, and quality of life.

### 3.6. PROMISE III

PROMISE III (NCT05313165) is a prospective, multicenter, single-arm study with an anticipated estimated enrollment of 100 patients. Primary outcome will be AFS to 6 months. Secondary outcomes include various measures, such as limb salvage, change in Rutherford classification, wound healing, and freedom from contrast-induced nephropathy, as well as procedural and technical success.

The purpose and status of the studies involving the treatment of CLTI with the LimFlow device are summarized in Table 2.

## 4. Discussion

Percutaneous DVA is considered the last-chance treatment for ‘no-option’ patients with CLTI. Palliative amputation, which is linked to a high risk of peri-procedural and 1-year mortality, is thereby potentially avoided. Due to the lack of reproducibility and standardization of the procedure, DVA has been attempted in multiple settings using tools and techniques currently available (i.e., ‘off-label’). In studies where alternate techniques of venous arterialization have been used, technical and clinical success varied from 77% to 100% and from 29% to 75%, respectively [32]. A multicenter retrospective study in Japan, the DEPARTURE JAPAN Study, reported the results of pDVA using the off-the-shelf technique. The VAST (venous arterialization simplified technique) was used in 83.3% of the cases. The technical success rate of pDVA using this technique was 88.9%, and the amputation-free survival rates at 6 and 12 months were 55.6% and 49.4%, respectively. Most of the studies evaluating DVA focus on AFS because it provides both a measure of the safety (mortality) and effectiveness (limb salvage) of the procedure [33]. Published studies demonstrate that pDVA using the LimFlow System can be performed with good technical success and high AFS rates at 6 and 12 months. More precisely, to date, according to the published data, technical success and AFS at 6 months is over 96% and 66%, respectively. This highlights that, whichever method is used to perform DVA, the right equipment (sheaths, wires, catheters, balloons, and stents) is crucial. With this approach, experts will be able to effectively manage possible complications and reduce the time of the procedure. A potential complication related to the pDVA procedure is bleeding at the vessel puncture when crossing is attempted with non-dedicated devices, which could lead to compartment syndrome. No complications were reported in the studies evaluating the LimFlow device [25]. However, rates of secondary interventions to optimize flow and wound healing are relatively high when compared with conventional arterial revascularization. This could be explained by the advanced and diffuse atherosclerotic disease of the target population [33]. Reinterventions can be endovascular or/and surgical and are reported for occlusion, asymptomatic stenosis, infection of the stent-graft, and bleeding from a superficial vein adjacent to the granulating wound. A variety of techniques and devices are used during reintervention, including thrombolysis, mechanical thrombectomy, DCB angioplasty, and stenting [32].

Challenges during pDVA using the LimFlow System include navigation through a complex pedal venous system and management of venous spasm. Pre-procedure ultrasound or venography contributes to identifying the venous anatomy and optimizing case planning [33]. Apart from that, the close monitoring of patients after the LimFlow procedure is mandatory, with prompt adjustments to postprocedural care, playing a key role in achieving good short-term and long-term results.

Finally, despite the upfront cost of the LimFlow System, the costs, prevalence, and mortality rate of CLTI qualify pDVA with the LimFlow System as a cost-effective and ‘high-value’ treatment compared to the status quo treatment [36].

### DVA Failure Types

Failures and subsequent reinterventions in the context of DVA are frequent occurrences. Venous lesions in particular tend to be more prevalent in cases of DVA failure and can ultimately lead to the failure of the DVA conduit. Whilst there are various models proposed to anticipate the occurrence and likelihood of restenosis or occlusion in DVA patients, a study by Zaman et al. introduced a model that illustrates the possibility of identifying restenosis patterns [37,38]. Their model offers a classification system that associates restenosis with specific anatomical locations and/or the positional relationship to the implanted devices. According to this classification, Type 1 failures correspond to occlusions impacting arterial inflow, encompassing lesions proximal to the DVA site (designated as 1a) or at the proximal stent edge of the DVA (identified as 1b). Type 2 pertains to occlusions occurring within the graft itself. Type 3 is characterized by occlusions affecting venous outflow, encompassing lesions at the DVA distal stent edge (categorized as 3a), lesions within the transitional veins (namely the medial/lateral plantar veins, denoted as 3b), or occlusions within previously placed stents in the transitional veins (represented as 3s). Type 4 encompasses lesions situated within the venous arch of the foot, specifically denoting the recurrent absence of filling in the entire pedal arch that had been present during the initial DVA completion. Type 5 cases remain ‘undefined’ due to the inability to precisely identify the location of the lesion(s) responsible for DVA failure. While distinct type and subtype patterns have been established, it is important to note that DVA failures may sometimes exhibit multiple failure types simultaneously. These findings play a critical role in guiding the creation of treatment strategies. 

To demonstrate the clinical relevance of their classification, Zaman et al. also outlined potential approaches for reintervention that are tailored to correspond with the occlusion patterns they observed [37]. To manage Type 1 failure patterns, their approach involved utilizing traditional balloon angioplasty or drug-coated balloon (DCB) angioplasty. In cases where there was an obstruction near the junction of the arteries and veins that could not be treated, retrograde access into the DVA stent-graft conduit was employed. This allowed the recreation of the connection between the proximal arteries and veins. Type 2 failures were addressed by performing PTA and, in some instances, an additional stent-graft was added to cover particularly persistent obstructions. 

For Type 3 lesions, the strategy involved PTA, extending the original stent-graft further downstream, or using a Supera woven nitinol bare-metal stent in the transitional vein. 

Type 4 failures were managed using PTA or a DCB, although the authors note that addressing obstructions in the venous arch can be quite challenging with standard catheter and wire techniques. Finally, the authors suggest that in cases where multiple types of failures coexist, a combination of treatment modalities may be deemed necessary, tailored to each specific lesion type.

## 5. Conclusions

The evolution of endovascular technology has allowed for the development of percutaneous deep vein arterialization (pDVA) approaches. The lack of options for CLTI patients highlights pDVA as a last option for limb salvage when amputation seems to be the only appropriate course. The advantage of pDVA is that it is minimally invasive in nature, which in turn permits the absence of wounds and lower rates of infections. The novel technique of the LimFlow device combines the advantages of surgical DVA with those of a minimally invasive procedure. Published data concerning the pDVA procedure with the LimFlow system demonstrate encouraging results concerning technical success, wound healing, and limb salvage. With the LimFlow device, vessel crossing, and valve disruption can be easily performed. Reinterventions are common due to the advanced and diffuse atherosclerotic disease of the target population. Long-term results can be achieved by optimizing the nutritive flow towards the ischemic tissue and through close postprocedural monitoring for any changes in the patient’s clinical condition (swelling, pain, or infection).

## Figures and Tables

**Table 1:** MI: myocardial infraction; PE: pulmonary embolism; DVT: deep vein thrombosis.

Advantages	Adverse Effects
Minimally invasive approach	Allergic reactions
Less chance of infection	Vascular complications (bleeding, hematoma)
Absence of wounds	Arterial and venous thromboembolic events (angina, stroke, limb ischemia, MI, PE, DVT)
	Contrast-induced nephropathy and renal failure
	Local or systemic infection
	Stent thrombosis, in-stent stenosis
	Unsuccessful disruption of valves

**Table 2:** Studies in the treatment of CLTI with the LimFlow device. (AFS: amputation-free survival).

Name	Purpose	Enrollment	AFS	Reintervention	Status
PROMISE I U.S.	To assess the safety, efficacy, and feasibility of the LimFlow system.	n = 32	91% (30-day) 74% (6-month) 70% (12-month)	n = 16 (52%)	Published April 2021 [35]
PROMISE II U.S.	To investigate the effectiveness and safety of the LimFlow System	n = 105	66.1% (6-month)	n = 38 (36.5%)	Published March 2023 [20]
PROMISE III U.S.	To provide additional data for the LimFlow system	n = 100			Ongoing
ALPS Study	To evaluate the midterm results of pDVA performed with the LimFlow device in no-option CLTI patients	n = 32	83.9% (6-month) 71% (12-month) 67.2% (24-month)	n = 17 (59.4%)	Published May 2018 [35]
PROMISE International	To evaluate the clinical effectiveness and safety of the LimFlow System	n = 35			Enrollment completed
PROMISE UK	To evaluate the clinical effectiveness and safety of the LimFlow System	n = 28			Enrollment completed

## Data Availability

Not applicable.
